# Functional characterization of a novel *CSF1R* mutation causing hereditary diffuse leukoencephalopathy with spheroids

**DOI:** 10.1002/mgg3.595

**Published:** 2019-02-06

**Authors:** Torsten Kraya, Dagmar Quandt, Thorsten Pfirrmann, Andrea Kindermann, Leonie Lampe, Matthias L. Schroeter, Jürgen Kohlhase, Dietrich Stoevesandt, Katrin Hoffmann, Pablo Villavicencio‐Lorini

**Affiliations:** ^1^ Department of Neurology Martin‐Luther‐University Halle‐Wittenberg Halle (Saale) Germany; ^2^ Institute of Anatomy and Cell Biology Martin‐Luther‐University Halle‐Wittenberg Halle (Saale) Germany; ^3^ Institute of Physiological Chemistry Martin‐Luther‐University Halle‐Wittenberg Halle (Saale) Germany; ^4^ Max-Planck Institute for Human Cognitive and Brain Sciences Leipzig Germany; ^5^ Clinic for Cognitive Neurology University Hospital Leipzig Germany; ^6^ SYNLAB Center for Human Genetics Freiburg Freiburg Germany; ^7^ Department of Radiology Martin‐Luther‐University Halle‐Wittenberg Halle (Saale) Germany; ^8^ Institute of Human Genetics Martin‐Luther‐University Halle‐Wittenberg Halle (Saale) Germany

**Keywords:** clinical diagnostics, disease, DNA, gene, molecular biology, mutation

## Abstract

**Background:**

Colony‐stimulating factor 1 receptor is a tyrosine kinase transmembrane protein that mediates proliferation, differentiation, and survival of monocytes/macrophages and microglia. *CSF1R *gene mutations cause hereditary diffuse leukoencephalopathy with spheroids (HDLS), an autosomal‐dominantly inherited microgliopathy, leading to early onset dementia with high lethality.

**Methods:**

By interdisciplinary assessment of a complex neuropsychiatric condition in a 44‐year old female patient, we narrowed down the genetic diagnostic to *CSF1R *gene sequencing. Flow cytometric analyses of uncultivated peripheral blood monocytes were conducted sequentially to measure the cell surface CSF1 receptor and autophosphorylation levels. Monocyte subpopulations were monitored during disease progression.

**Results:**

We identified a novel heterozygous deletion–insertion mutation c.2527_2530delinsGGCA, p.(Ile843_Leu844delinsGlyIle) in our patient with initial signs of HDLS. Marginally elevated cell surface CSF1 receptor levels with increased Tyr723 autophosphorylation suggest an enhanced receptor activity. Furthermore, we observed a shift in monocyte subpopulations during disease course.

**Conclusion:**

Our data indicate a mutation‐related CSF1R gain‐of‐function, accompanied by an altered composition of the peripheral innate immune cells in our patient with HDLS. Since pharmacological targeting of CSF1R with tyrosine kinase inhibitors prevents disease progression in mouse models of neurodegenerative disorders, a potential pharmacological benefit of CSF1R inhibition remains to be elucidated for patients with HDLS.

## 1 INTRODUCTION

Hereditary diffuse leukoencephalopathy with spheroids [HDLS, MIM#221820] is an autosomal‐dominant neurodegenerative disease characterized by white matter changes and axonal deterioration causing rapid decline of cognitive‐ and motor functions, as well as personality changes (Axelsson, Roytta, Sourander, Akesson, & Andersen, 1984). Hereditary diffuse leukoencephalopathy with spheroids is summarized together with pigmented orthochromatic leukodystrophy (POLD) as adult‐onset leukoencephalopathy with axonal spheroids and pigmented glia (ALSP) (Wider et al., 2009). Clinical delineation of this entity is challenging due to variable clinical presentations reminding of Alzheimer´s disease (Sundal et al., 2012), multiple sclerosis (Inui et al., 2013; Sundal et al., 2015), cerebral arteriopathy with subcortical infarcts and leukoencephalopathy (CADASIL) (Kleinfeld et al., 2013), Parkinson´s disease (Lynch et al., 2016), or frontotemporal dementia (Rademakers et al., 2012; Sundal et al., 2012).

Colony‐stimulating factor 1 receptor [*CSF1R*, MIM*164770] mutations cause HDLS (Rademakers et al., 2012). CSF1R is a tyrosine kinase transmembrane protein involved in activation of mononuclear phagocytic cells, for example, microglia that function as immune effector cells with homeostatic and surveillance tasks in the brain (Prinz & Priller, 2014). As microglial dysfunction due to *CSF1R* mutation is assumed to be the primary disease‐causing mechanism, HDLS is classified as microgliopathy (Sasaki, 2017). Most of the mutations are located in the tyrosine kinase domain of CSF1R and are discussed to cause CSF1R loss‐of‐function (Konno, Kasanuki, Ikeuchi, Dickson, & Wszolek, 2018; Pridans, Sauter, Baer, Kissel, & Hume, 2013; Rademakers et al., 2012). Recently, a clinical and genetic comparison of 122 cases from 90 families with *CSF1R* mutations revealed no apparent genotype–phenotype correlation but showed a gender‐dependent preponderance with a significant younger age of onset in women than men (Konno et al., 2017). The mean onset of manifestation in females and males together was 43 years and the mean disease survival 6.8 years.

Monocytes are a heterogeneous group of cells belonging to innate immunity and like microglia they are part of the mononuclear phagocytic system (Katsumoto, Takeuchi, Takahashi, & Tanaka, 2018). There is consensus about the existence of at least three different blood monocyte subpopulations (Murray, 2018; Sampath, Moideen, Ranganathan, & Bethunaickan, 2018). Classical, intermediate, and nonclassical monocytes are recognized by differential cell surface marker patterns and divergent transcriptomic and proteomic profiles (Wong et al., 2011; Ziegler‐Heitbrock, 2015). Of note, monocytes of the nonclassical subpopulation show the highest CSF1R levels (Wong et al., 2011). In particular, the regulation, distribution, and ligand binding capacity of CSF1R along different mononuclear phagocytes in diverse tissues is not fully understood yet (Herz, Filiano, Smith, Yogev, & Kipnis, 2017). Two ligands for CSF1R, CSF1 and IL34, have been identified. Microglia cells certainly do depend on CSF1R, but CSF1R transgene reporter mice systems revealed a heterogeneous expression within microglia with particular lower transgene expression in the cerebellum as compared to other regions of the brain (Hawley et al., 2018). Furthermore, there is evidence for other components in addition to CSF1R involved in the self‐renewal of the brain including microglia, such as the IL1R pathway (Bruttger et al., 2015). Interestingly, although the original microglia in the brain are not derived from circulating monocytes but from yolk sac, in conditions of inflammatory brain disorders, monocytes from the periphery enter the brain and take part in the repopulation of microglia (Askew et al., 2017). These aforementioned facts about the role of CSF1R in monocyte/macrophages as well as in microglia biology suggest a fundamental impact and therapeutic potential for HDLS.

## 2 MATERIALS AND METHODS

### 2.1 Editorial policies and ethical considerations

This study was approved by the local ethics committee and was performed in accordance with the Declaration of Helsinki. The patient gave a written informed consent for the scientific use and publication of medical records and genetic results.

### 2.2 *Brain* magnetic resonance imaging

Initial imaging was performed using a 1.5‐Tesla MR machine (Magnetom Sonata Vision, Siemens). For follow‐up scans, we used the aforementioned MR machine and a 3‐Tesla MR machine (Magnetom Verio, Siemens). Cerebral magnetic resonance imaging included T1‐weighted (T1 SE, T1‐VIBE), T2‐FLAIR‐weighted, and diffusion imaging sequences.

### 2.3 Samples

DNA and PBMC of the patient as well as age‐ and gender‐matched healthy controls were obtained from EDTA peripheral blood samples. PBMC were gained by Ficoll gradient. Total blood and major immune cell population count were obtained by the use of a hematology analyzer (Cell Dyn, Abbott).

### 2.4 Gene sequencing

PCR amplification and Sanger sequencing of all protein coding exons and ±20 bp flanking intronic regions of the *CSF1R *gene were performed according to standard protocols. Used oligonucleotide primers for *CSF1R* gene sequencing can be depicted form the supplemental material. Sequence analysis was performed on an ABI3130xl capillary sequencer (Applied Biosystems). Sequence data were processed using ABI software and were analyzed using Sequence Pilot (JSI medical systems GmbH) based on the complementary DNA (cDNA) reference sequence for *CSF1R* (RefSeq NM_005211.3, NC_000005.10). Prediction of pathogenicity of the variant was performed by the in silico programs MutationTaster (Schwarz, Cooper, Schuelke, & Seelow, 2014) and PROVEAN (Choi & Chan, 2015). The sequence variant description was checked using the Mutalyzer program (den Dunnen et al., 2016). The identified mutation was submitted to the public database ClinVar http://www.ncbi.nlm.nih.gov/clinvar/ (accession number SCV000787829). For graphical overview of *CSF1R* mutations, known pathogenic/likely pathogenic variants were downloaded from ClinVar database and plotted using the MutationMapper tool (Cerami et al., 2012; Gao et al., 2013).

### 2.5 Protein structure model

Protein structure files were downloaded from PDB (https://doi.org/10.2210/pdb4R7H/pdb) (Tap et al., 2015). Files were processed with PyMOL and the mutation p.(Ile843_Leu844delinsGlyIle) inserted in the secondary structure. The protein structures are displayed as cartoon with amino acid side chains at the respective mutation sites.

### 2.6 Flow cytometry

Flow cytometric analyses of surface receptor levels on live cells and of intracellular components on fixed/permeabilized cells were essentially performed as recently described (Pfirrmann et al., 2015; Quandt, Fiedler, Boettcher, Marsch, & Seliger, 2011). Briefly, 1 x 10^5^ cells were stained with specific antibodies in FACS buffer (PBS with 1% FCS and 0.3 mmol/L EDTA). Prior to specific antibody staining with anti‐CSF1R (CD115, clone 9‐4D2‐1E4, BioLegend, USA) and anti‐CD14 (clone TÜK4, Miltenyi Biotec, Germany) on live cells, treatment with Fcγ block for 10 min on ice was performed. For live cell analyses, dead cells were excluded using PI staining. For intracellular analyses, cells were fixed for 10 min at 37°C with 4% paraformaldehyde. Followed by two washing steps, cells were permeabilized with 90% methanol buffer for 30 min on ice. Prior to specific antibody staining with anti‐phospho‐CSF1R (Tyr723) (clone 49C10, Cell Signaling, USA) and F(ab’)2 goat anti‐rabbit IgG‐Alexa488 (polyclonal, A11070, ThermoFisher) in FACS buffer, cells were incubated with Fcγ block for 15 min. Flow cytometry was performed using a LSR Fortessa^TM ^(Becton Dickinson) flow cytometer and FACS‐Diva^TM^ and FlowJo^TM ^(Tree Star) software. To compare CSF1R surface levels between individuals or different blood donations of the patient, we used median fluorescence intensities values. Delta median fluorescence intensities, meaning subtraction of background median obtained by sole secondary antibody staining, were used to display intracellular phospho‐CSF1R levels. Statistical analysis was performed using Graphpad Prism 7.0, and a Student’s *t* test or Mann–Whitney *U* test was applied whereat *p* < 0.05* was considered significant. Data are summarized in floating bars with line at mean.

## 3 RESULTS

### 3.1 Case report

**Figure 1:**
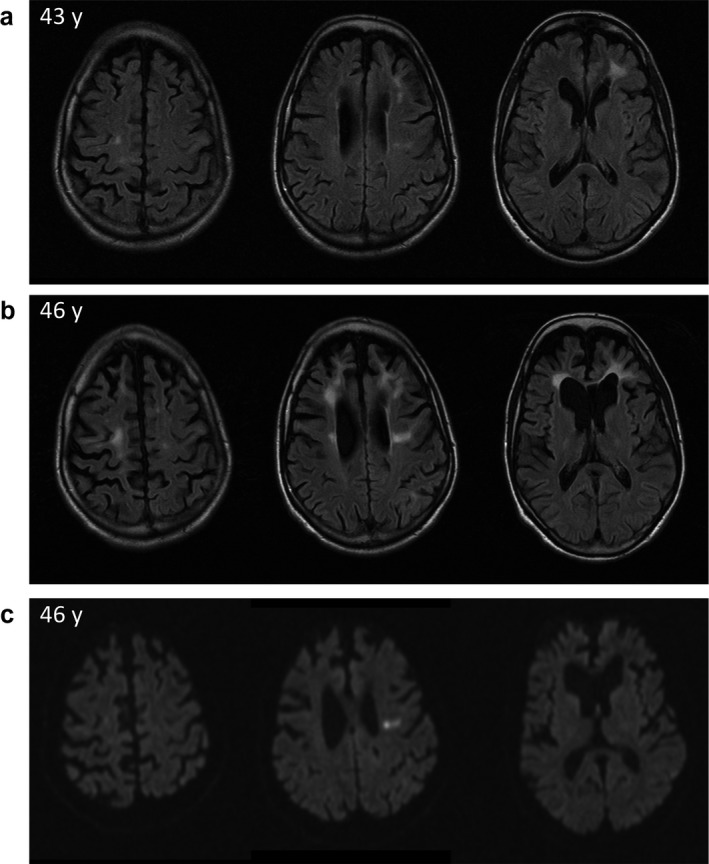
Magnetic resonance imaging (MRI) of the brain. (a) Initial diffuse white matter lesions and gliosis at the age of 43 years. (b) Follow‐up MRI at age of 46 years showing increasing diffuse white matter lesions and progression of frontotemporal cerebral atrophy. (c) Diffusion weighted imaging (DWI) of the follow‐up examination revealing various isolated spots of hyperintense signal as sign of diffusion restriction and characteristic imaging feature for HDLS

**Figure 2:**
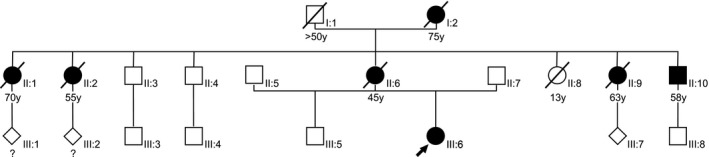
Family pedigree. The accumulation of different neurological disorders over three generations reflects an autosomal dominant inheritance pattern with phenotypic variability.** (**I:2) polyneuropathy, kidney disease, and diabetes mellitus; (II:1) multiple cerebral infarctions; (II:2) mobilization with walking frame, history of thrombosis, and alcohol abuse; (II:6) aphasia and paresis after cerebral infarction at age of 38; alcohol abuse, smoker, elevated arterial blood pressure; (II:9) clinical diagnosis of Parkinson due to gait disturbance, aphasia, epilepsy; (II:10) cerebral infarction; (III:6) index patient

The 43‐year‐old female patient was initially referred to our university hospital because of progressive psychomotor decline during a period of about 1 year. Since the initial magnetic resonance imaging (MRI) of the brain revealed symmetric atrophy pronounced in the frontal lobes and periventricular with matter lesions a neurological examination was initiated (Figure 1a). The complex clinical presentation including progressive spastic‐ataxic gait, spastic hemiparesis, apraxia, hand tremor, saccadic eye movements, speech production disorder, and brisk tendon reflexes was topologically correlated with the brain MRI alterations. For further differential diagnostics of an assumed inherited microangiopathy, the patient was referred to our genetic department at age of 44 years. No dysmorphological features suggesting a recognizable syndrome were detected. The pedigree analysis over three generations revealed several affected relatives with neurological disorders, indicating an autosomal dominant mode of inheritance (Figure 2). The patient herself had no children. As far as known, the patient's mother had passed away at age of 45 years because of cerebral infarction leading to rapid neurological decline with aphasia and paralysis. A maternal aunt of the patient had died after several years of tentative diagnosis of Parkinson´s disease. Two maternal uncles of the patient were also supposed to have cerebral infarctions, one of them already deceased. The maternal grandmother is said to have died by renal insufficiency and polyneuropathy in association with diabetes mellitus. On several cousins, no information on their health conditions was available.

Differential diagnoses including CADASIL (cerebral arteriopathy, autosomal dominant, with subcortical infarcts, and leukoencephalopathy), Fabry disease, Alzheimer's as well as Parkinson's disease, and frontotemporal dementia were considered clinically. However, using OMIM database search and the program Phenomizer (Kohler et al., 2009, 2017), we found the best congruence with the clinical synopsis of HDLS and initiated targeted gene analysis of *CSF1R* which allowed us to confirm the diagnosis of HDLS in our patient.

During follow‐up care, our patient was thoroughly examined by a neuropsychologist and a patholinguist at age of 46 years. In accordance with the literature (Freeman et al., 2009; Kohler, Curiel, & Vanderver, 2018), testing revealed rather unspecific cognitive deficits with a score of 28 of 30 points in the Mini Mental State Examination (MMSE). Impairment was proven in selective and divided attention, executive functions, and delayed recall in memory. The speech therapeutic diagnostics revealed hypokinetic dysarthria rather than aphasia as the patient was not able to speak, because phonation and word production were so difficult for her. Her understanding was actually quite well. In written form, she was able to produce grammatically correct sentences with orthographically challenging wording. The neuropsychiatric inventory (Schroeter et al., 2011) revealed apathy and depressive symptoms, the latter especially when she was confronted with her disease. In the neurological follow‐up examination, 1 month later, the patient showed further progressive psychomotor decline with severe gait bradykinesia, postural instability, and spastic tetraparesis. The speech therapy had led to slight improvement of the hypokinetic dysarthria. However, severely impeded communication skills and labile affect were still present.

Follow‐up MRI scans 30–34 months after the initial MRI imaging revealed a drastic progression of leukodystrophy with patchy and confluent bilateral white matter hyperintensities predominantly in the frontal and prefrontal white matter (Figure 1b,c; Figure S1). Slightly less intense white matter changes with heterogeneous pattern were also seen in the bilateral parietal white matter. The configuration of some of the changes were tract‐shaped and along the corticospinal tract bilaterally. We observed an asymmetry with right‐sided accentuation of the bilateral ventricular dilatation as a sign of subcortical atrophy. Focal diffusion restrictions were seen in the bilateral precentral white matter as a correlate of the active inflammatory and degenerative processes as described characteristic for HDLS (Bender et al., 2014). A pronounced thinning of the corpus callosum and a diffuse cortical atrophy was observed.

### 3.2 Molecular genetics

**Figure 3:**
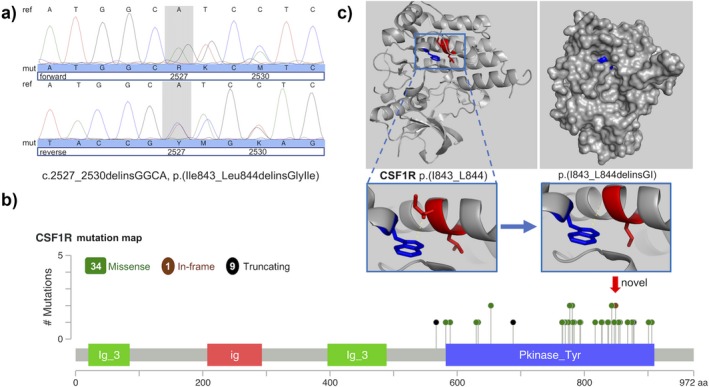
Molecular etiology. (a) Result of the genetic analysis of the patient revealing the heterozygous sequence variant c.2527_2530delinsGGCA, p.(Ile843_Leu844delinsGlyIle) in exon 19 of the *CSF1R* gene (NM_005211.3). (b) Mutation map of previously described *CSF1R* mutations. (c) Protein structural analysis of the CSF1R region containing the tyrosine kinase domain comparing wild‐type and mutated state

By direct Sanger sequencing of *CSF1R*, we identified in exon 19 the heterozygous sequence variant c.2527_2530delinsGGCA, p.(Ile843_Leu844delinsGlyIle) (Figure 3a). As shown in the mutation map of previously described *CSF1R* mutations, the identified variant of our patient represents a novel variant located in a mutation hotspot (Figure 3b). The variant is predicted to be disease causing by the in silico program MutationTaster, since the amino acids at codons 843 and 844 belong to the tyrosine kinase domain of the CSF1 receptor and are highly conserved. The prediction tool PROVEAN classified the variant also as deleterious. Additional information on the prediction results are documented in the supplemental material according to the guidelines for reporting and using prediction tools for genetic variation analysis (Vihinen, 2013). Segregation analysis of the variant was not possible due to deceased or unreachable family members. Therefore, we focused on structural model analysis of the CSF1R showing that the amino acid residues 843 and 844 (highlighted in red) are part of an α‐helix secondary structure buried within the molecule without surface exposure (Figure 3c). Neighboring residues within the range of 3.5 Ångström (Å) are present between amino acid Ile843 (red) and Trp821 (blue). The interaction of the corresponding amino acid side chains is highlighted by a yellow line, as shown by magnification of the region. The mutation results in a loss of interaction between Ile843 and Trp821.

### 3.3 Functional analysis

**Figure 4:**
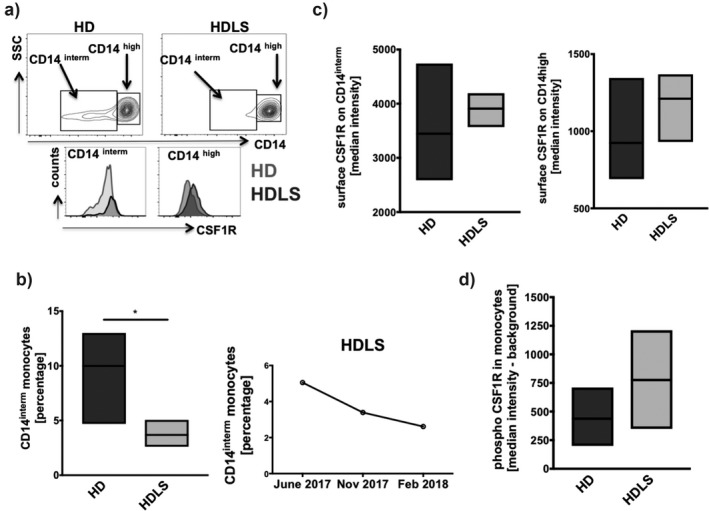
Peripheral monocyte subtype distribution and CSF1R surface expression and constitutive intracellular phospo‐CSF1R in HDLS. Gender‐ and age‐matched healthy donors (HD) were used for comparison (*n* = 5). HDLS patient blood was obtained at three time points. Density gradient‐derived PBMC were used for analysis. (a) Example FACS plots are given for CD14^high^ and CD14^interm^ monocyte distribution and for surface CSF1R expression divided for monocyte subpopulations (CD14^high^ and CD14^interm^) for HDLS and HD. (b) Summary of monocyte subpopulation of CD14^interm^ is given as combined values or as single values for the HDLS patient over time. (c) Surface expression of CSF1R on CD14^high^ and CD14^interm ^expressing monocytes was measured and is plotted as median intensity. (d) Intracellular phospho‐CSF1R expression on total CD14 monocytes upon fixation and methanol permeabilization was analyzed directly from PBMC ex vivo. Delta median intensity values with background subtraction are given. Data are obtained by flow cytometry and cells of (a–c) are gated on live cells by propidium iodide exclusion. Floating bars from Min to Max are given and significance refers to *p* < 0.05

To investigate the functional effect of the *CSF1R* variant in the mononuclear phagocytic system of our HDLS patient, we directly analyzed peripheral blood mononuclear cells (PBMCs) from three independent samples of different time points by flow cytometry. For comparison, we used five age‐ and gender‐matched healthy donors (HD) analyzed on different days along with the patient or separately. To dissect different monocyte subpopulations, we stained PBMCs with anti‐CD14 antibodies and occasionally with anti‐CD16 antibodies. CD14^interm^CD16^pos ^monocytes belong to nonclassical whereas CD14^high ^expressing monocytes harbor classical (CD14^high^CD16^neg^) as well as intermediate (CD14^high^CD16^pos^) monocytes. Our data distinguish two monocyte populations on the basis of CD14 expression and monocyte gates in all individual experiments. Example FACS plots illustrating CSF1R surface levels on nonclassical CD14 intermediate and on CD14 high expressing monocytes are depicted on Figure 4a. The summary of the surface CSF1R expression is given in Figure 4c and illustrates the before identified higher expression of CSF1R on nonclassical monocytes in both HD (3,447 ± 406) and HDLS (3,909 ± 183) as compared to classical monocytes HD (924 ± 120) and HDLS (1,211 ± 141). Additionally, we found a small trend toward higher expression of CSF1R on both classical and nonclassical monocytes of the HDLS patient (Figure 4c). Interestingly, at the same time, we found a significantly decreased frequency of nonclassical monocytes in the HDLS patient as compared to HD (Figure 4b). This loss of nonclassical monocytes occurred over time of the disease course. At all time points of blood withdrawal, the patient had normal total white blood cell counts without signs of infection. However, total monocyte counts were increasing, whereas lymphocyte counts were decreasing over time of the disease course (Figure S2). Ex vivo intracellular analysis of autophosphorylated CSF1R as means of functional read‐out revealed moderate increased median intensities of 776 ± 249 for the HDLS patient as compared to HD with 437 ± 114 (Figure 4d).

## 4 DISCUSSION

Hereditary diffuse leukoencephalopathy with spheroids is an autosomal dominant neurodegenerative disorder that typically presents with broad phenotypic variability (Sundal et al., 2012). Most of the patients with HDLS are diagnosed postmortem or after stereotactic brain biopsy with typical histological findings of gliosis and axonal spheroids (Kortvelyessy et al., 2015; Rademakers et al., 2012). The white matter lesions can affect different parts of the central nervous system and cause variable neurological symptoms (Konno et al., 2017; Kortvelyessy et al., 2015). As described previously (Sundal & Wszolek, 1993), the following clinical constellation is a sufficient indication for molecular genetic testing of HDLS: First, manifestation at mid‐adulthood with rapidly progressive neurological decline including gait, speech and behavioral changes. Second, dispersed white matter changes with signs of gliosis. Third, positive family history for different neuropsychiatric disorders reminding of CADASIL, multiple sclerosis, Parkinson’s, Alzheimer’s, or Fabry disease. Recently, further diagnostic criteria with high sensitivity and sufficient specificity for differentiation from other leukoencephalopathies have been established (Konno, Yoshida et al., 2018). Regarding the different genetic diagnostic methods, for example gene panel analysis of various leukoencephalopathy‐associated genes versus clinically guided single gene sequencing one has to consider that a direct approach reduces incidental findings or unclassified sequence variants and facilitates a purposeful genetic counselling. In turn, a gene panel approach might be more cost and time efficient depending on the clinical presetting (Lynch et al., 2018).

The clinical findings of our patient complied with above mentioned diagnostic criteria and led to the identification of a novel *CSF1R* mutation by single gene sequencing. Segregation analysis was not feasible due to deceased or unreachable relatives of our patient. However, the pedigree is highly suspicious for an autosomal dominant inheritance of HDLS with overlap to findings of Parkinson’s disease, CADASIL, and Fabry disease. Regular clinical monitoring of the patient revealed rapidly progressive psychomotor disturbances in correlation with brain imaging alterations, which is a well‐known course in leukodystrophies, especially in HDLS (Konno et al., 2017).

CSF1R is a critical mediator of microglial function (Konno, Kasanuki et al., 2018) and regulates microglia density and distribution in the brain (Oosterhof et al., 2018). Most of the previously identified HDLS‐associated *CSF1R* mutations are missense or splice‐site variants leading to amino acid changes in the tyrosine kinase domain and only a few are located in other protein domains (Konno, Kasanuki et al., 2018; Rademakers et al., 2012). The identified amino acid changes of the CSF1 receptor of our patient are located in the tyrosine kinase domain within an α‐helix structure without surface accession. We reason that p.(Leu844Ile) plays a minor role because of similar chemical and structural characteristics of leucine and isoleucine. Instead, p.(Ile843Gly) is likely disease‐causing for several reasons. First, glycine residues are known to destabilize α‐helical structures (Serrano, Neira, Sancho, & Fersht, 1992). Second, isoleucine at position 843 seems to be essential, as the two pathogenic mutations p.(Ile843Asn) and p.(Ile843Phe) had been reported in patients with HDLS before (Battisti et al., 2014; Karle et al., 2013). Third, the importance of this α‐helical structure is further supported by the description of the mutations p.(Asp837Tyr) and p.(Phe849Ser) causing HDLS (Rademakers et al., 2012). However, these mutations have not been characterized functionally yet.

So far, in vitro analyses of other *CSF1R* mutations using transiently transfected HeLa or Ba/F3 cell lines resulted either in absent autophosphorylation or in failure to maintain CSF1 dependent cell survival (Pridans et al., 2013; Rademakers et al., 2012). However, in blood and brain samples CSF1R phosphorylation was not altered (Rademakers et al., 2012). Since mutant CSF1 receptors can be expressed on the cell surface but hamper CSF1‐dependent signaling, a dominant negative disease mechanism has been postulated (Pridans et al., 2013; Rademakers et al., 2012). This complies with observations that only multiple combined autophosphotyrosine mutations or simultaneous inhibition of three downstream signaling pathways completely block induction of D2 in response to CSF‐1 (Dey et al., 2000). In turn, based on reduced CSF1R levels in a patient´s brain sample with a *CSF1R* frameshift mutation, haploinsufficiency is discussed as an alternative molecular mechanism (Konno et al., 2014). However, as shown in their supplemental data, it seems that an antibody against the *N*‐terminus (B‐8) is more suitable to discard a truncated mutant protein that might interfere with the full‐length wild‐type protein.

We analyzed blood‐derived monocytes for cell surface expression and phosphorylation of CSF1R by flow cytometry. The mutated CSF1 receptor our patient seems to be at least functional in phosphorylation, and thus dimerization and the increased phosphorylation indicates an apparent gain‐of‐function. We speculate that the amino acid changes in the α‐helical region either result in disruption of surface structures important to bind proteins involved in CSF1R degradation, or in locking the enzyme in a conformational state unable to do so. Even mutant homo‐ and heterodimers with a presumed gain‐of‐function on the receptor level might override the normal receptor function by perturbing downstream signaling dominant‐negatively causing cell‐specific pleiotropic impacts (De et al., 2014; Dey et al., 2000; Hamilton, 1997). As we do not know whether this finding is mutation specific, other HDLS causing *CSF1R* mutations should be analyzed in primary immune cells.

*Csf1r* null mutant mice reveal severe osteopetrosis due to deficient osteoclast activation (Dai et al., 2002; Li, Chen, Zhu, & Pollard, 2006) and structural brain abnormalities with impact on microglia and neural progenitor cells (Erblich, Zhu, Etgen, Dobrenis, & Pollard, 2011; Nandi et al., 2012), but it was not possible to proof neurodegeneration because of early onset high lethality. However, these obvious developmental defects match well with a congenital disorder of two deceased infants assumed to carry a homozygous *CSF1R* nonsense mutation (Monies et al., 2017). Since the parental heterozygous carrier status was harmless CSF1R loss‐of‐function mutations seem to display recessive alleles associated with congenital developmental disorders and not dominant alleles causing HDLS by haploinsufficiency (Monies et al., 2017). Moreover, HDLS patients do neither exhibit osteopetrosis nor any other bone structure abnormalities (Rademakers et al., 2012). On the other hand, a mutant *Csf1r* mouse strain with a haploinsufficient allele resembled HDLS‐like symptoms (Chitu et al., 2015), but some neuropathological findings differed from patients with HDLS (Konno, Kasanuki et al., 2018). Recently, *Csf1r* knockout rats were established, but, although they survive well into adulthood, they do not reveal an overt phenotype in brain despite the complete absence of microglia (Pridans et al., 2018). The authors suggest that the impact of the loss of neuroprotective function of microglia in the *Csf1r* knockout rat may be mitigated by the absence of monocytes. Interestingly, recruitment of hematogenous macrophages into the CNS is assumed to take place in patients with HDLS (Tada et al., 2016). Therefore, we focused on the analysis of circulating primary immune cells known to depend on CSF1R signaling. Similar as described earlier (Wong et al., 2011), we found a differential expression of CSF1R on primary monocyte subpopulations of healthy donors and HDLS with the highest levels on nonclassical monocytes. Of particular note, we found a sequential loss of nonclassical monocytes in the HDLS patient over time whereas the total blood monocyte count was increased. Under circumstances of glucocorticoid treatment, the same phenomenon was found where a selective reduction of nonclassical monocytes by apoptosis induction was identified as responsible mechanism (Dayyani et al., 2003). However, the herein investigated HDLS patient did not take glucocorticoids. The absence of nonclassical monocytes in the blood has been identified before in three siblings, where one diseased and two were healthy. In all three cases, no genetic alteration in *CSF1* or *CSF1R* was found (Frankenberger et al., 2013). However, our observation of CSF1R dysfunction along with highest expression in nonclassical monocytes raises the question whether these monocytes invade into cerebral tissue and whether their removal from blood might be beneficial for HDLS patients. Interestingly, hematopoietic stem cell transplantation halted the disease progression in a patient with HDLS (Eichler et al., 2016). Furthermore, it remains to be elucidated whether levels of CSF1 and IL‐34 in the plasma and cerebrospinal fluid as well as ex vivo* CSF1R* functional studies of patient‐derived monocytes might serve to investigate a potential pharmacological benefit of CSF1R inhibition in patients with HDLS. This approach is motivated by observations that CSF1R tyrosine kinase inhibitors prevent disease progression in mouse models of Alzheimer´s disease and amyotrophic lateral sclerosis, among others by reducing invasion of macrophages (Martinez‐Muriana et al., 2016; Olmos‐Alonso et al., 2016). However, one has to consider that microglia and monocyte response could be both neuroprotective and neurotoxic depending on the stage and progression of the disease (Baufeld, O'Loughlin, Calcagno, Madore, & Butovsky, 2018).

## CONFLICT OF INTEREST

None declared.

## References

[mgg3595-bib-0001] Askew, K. , Li, K. , Olmos‐Alonso, A. , Garcia‐Moreno, F. , Liang, Y. , Richardson, P. , … Gomez‐Nicola, D. (2017). Coupled proliferation and apoptosis maintain the rapid turnover of microglia in the adult brain. Cell Reports, 18(2), 391–405. 10.1016/j.celrep.2016.12.041 28076784 PMC5263237

[mgg3595-bib-0002] Axelsson, R. , Roytta, M. , Sourander, P. , Akesson, H. O. , & Andersen, O. (1984). Hereditary diffuse leucoencephalopathy with spheroids. Acta Psychiatrica Scandinavica, 314, 1–65. 6595937

[mgg3595-bib-0003] Battisti, C. , Di Donato, I. , Bianchi, S. , Monti, L. , Formichi, P. , Rufa, A. , … Federico, A. (2014). Hereditary diffuse leukoencephalopathy with axonal spheroids: Three patients with stroke‐like presentation carrying new mutations in the CSF1R gene. Journal of Neurology, 261(4), 768–772. 10.1007/s00415-014-7257-3 24532199

[mgg3595-bib-0004] Baufeld, C. , O'Loughlin, E. , Calcagno, N. , Madore, C. , & Butovsky, O. (2018). Differential contribution of microglia and monocytes in neurodegenerative diseases. Journal of Neural Transmission, 125(5), 809–826. 10.1007/s00702-017-1795-7 29063348 PMC7255107

[mgg3595-bib-0005] Bender, B. , Klose, U. , Lindig, T. , Biskup, S. , Nagele, T. , Schols, L. , & Karle, K. N. (2014). Imaging features in conventional MRI, spectroscopy and diffusion weighted images of hereditary diffuse leukoencephalopathy with axonal spheroids (HDLS). Journal of Neurology, 261(12), 2351–2359. 10.1007/s00415-014-7509-2 25239393

[mgg3595-bib-0006] Bruttger, J. , Karram, K. , Wortge, S. , Regen, T. , Marini, F. , Hoppmann, N. , … Waisman, A. (2015). Genetic cell ablation reveals clusters of local self‐renewing microglia in the mammalian central nervous system. Immunity, 43(1), 92–106. 10.1016/j.immuni.2015.06.012 26163371

[mgg3595-bib-0007] Cerami, E. , Gao, J. , Dogrusoz, U. , Gross, B. E. , Sumer, S. O. , Aksoy, B. A. , … Schultz, N. (2012). The cBio cancer genomics portal: An open platform for exploring multidimensional cancer genomics data. Cancer Discovery, 2(5), 401–404. 10.1158/2159-8290.CD-12-0095 22588877 PMC3956037

[mgg3595-bib-0008] Chitu, V. , Gokhan, S. , Gulinello, M. , Branch, C. A. , Patil, M. , Basu, R. , … Stanley, E. R. (2015). Phenotypic characterization of a Csf1r haploinsufficient mouse model of adult‐onset leukodystrophy with axonal spheroids and pigmented glia (ALSP). Neurobiology of Disease, 74, 219–228. 10.1016/j.nbd.2014.12.001 25497733 PMC4323933

[mgg3595-bib-0009] Choi, Y. , & Chan, A. P. (2015). PROVEAN web server: A tool to predict the functional effect of amino acid substitutions and indels. Bioinformatics, 31(16), 2745–2747. 10.1093/bioinformatics/btv195 25851949 PMC4528627

[mgg3595-bib-0010] Dai, X. M. , Ryan, G. R. , Hapel, A. J. , Dominguez, M. G. , Russell, R. G. , Kapp, S. , … Stanley, E. R. (2002). Targeted disruption of the mouse colony‐stimulating factor 1 receptor gene results in osteopetrosis, mononuclear phagocyte deficiency, increased primitive progenitor cell frequencies, and reproductive defects. Blood, 99(1), 111–120. 10.1182/blood.V99.1.111 11756160

[mgg3595-bib-0011] Dayyani, F. , Belge, K. U. , Frankenberger, M. , Mack, M. , Berki, T. , & Ziegler‐Heitbrock, L. (2003). Mechanism of glucocorticoid‐induced depletion of human CD14+CD16+ monocytes. Journal of Leukocyte Biology, 74(1), 33–39. 12832440 10.1189/jlb.1202612

[mgg3595-bib-0012] De, I. , Nikodemova, M. , Steffen, M. D. , Sokn, E. , Maklakova, V. I. , Watters, J. J. , & Collier, L. S. (2014). CSF1 overexpression has pleiotropic effects on microglia in vivo. Glia, 62(12), 1955–1967. 10.1002/glia.22717 25042473 PMC4205273

[mgg3595-bib-0013] den Dunnen, J. T. , Dalgleish, R. , Maglott, D. R. , Hart, R. K. , Greenblatt, M. S. , McGowan‐Jordan, J. , … Taschner, P. E. (2016). HGVS recommendations for the description of sequence variants: 2016 update. Human Mutation, 37(6), 564–569. 10.1002/humu.22981 26931183

[mgg3595-bib-0014] Dey, A. , She, H. , Kim, L. , Boruch, A. , Guris, D. L. , Carlberg, K. , … Li, W. (2000). Colony‐stimulating factor‐1 receptor utilizes multiple signaling pathways to induce cyclin D2 expression. Molecular Biology of the Cell, 11(11), 3835–3848. 10.1091/mbc.11.11.3835 11071910 PMC15040

[mgg3595-bib-0015] Eichler, F. S. , Li, J. , Guo, Y. , Caruso, P. A. , Bjonnes, A. C. , Pan, J. , … Saxena, R. (2016). CSF1R mosaicism in a family with hereditary diffuse leukoencephalopathy with spheroids. Brain, 139(Pt 6), 1666–1672. 10.1093/brain/aww066 27190017 PMC4892751

[mgg3595-bib-0016] Erblich, B. , Zhu, L. , Etgen, A. M. , Dobrenis, K. , & Pollard, J. W. (2011). Absence of colony stimulation factor‐1 receptor results in loss of microglia, disrupted brain development and olfactory deficits. PLoS ONE, 6(10), e26317 10.1371/journal.pone.0026317 22046273 PMC3203114

[mgg3595-bib-0017] Frankenberger, M. , Ekici, A. B. , Angstwurm, M. W. , Hoffmann, H. , Hofer, T. P. , Heimbeck, I. , … Ziegler‐Heitbrock, L. (2013). A defect of CD16‐positive monocytes can occur without disease. Immunobiology, 218(2), 169–174. 10.1016/j.imbio.2012.02.013 22459269

[mgg3595-bib-0018] Freeman, S. H. , Hyman, B. T. , Sims, K. B. , Hedley‐Whyte, E. T. , Vossough, A. , Frosch, M. P. , & Schmahmann, J. D. (2009). Adult onset leukodystrophy with neuroaxonal spheroids: Clinical, neuroimaging and neuropathologic observations. Brain Pathology, 19(1), 39–47. 10.1111/j.1750-3639.2008.00163.x 18422757 PMC2757058

[mgg3595-bib-0019] Gao, J. , Aksoy, B. A. , Dogrusoz, U. , Dresdner, G. , Gross, B. , Sumer, S. O. , … Schultz, N. (2013). Integrative analysis of complex cancer genomics and clinical profiles using the cBioPortal. Science Signalling, 6(269), pl1 10.1126/scisignal.2004088 PMC4160307 23550210

[mgg3595-bib-0020] Hamilton, J. A. (1997). CSF‐1 signal transduction. Journal of Leukocyte Biology, 62(2), 145–155. 10.1002/jlb.62.2.145 9261328

[mgg3595-bib-0021] Hawley, C. A. , Rojo, R. , Raper, A. , Sauter, K. A. , Lisowski, Z. M. , Grabert, K. , … Jenkins, S. J. (2018). Csf1r‐mApple transgene expression and ligand binding in vivo reveal dynamics of CSF1R expression within the mononuclear phagocyte system. Journal of Immunology, 200(6), 2209–2223. 10.4049/jimmunol.1701488 PMC5834790 29440354

[mgg3595-bib-0022] Herz, J. , Filiano, A. J. , Smith, A. , Yogev, N. , & Kipnis, J. (2017). Myeloid cells in the central nervous system. Immunity, 46(6), 943–956. 10.1016/j.immuni.2017.06.007 28636961 PMC5657250

[mgg3595-bib-0023] Inui, T. , Kawarai, T. , Fujita, K. , Kawamura, K. , Mitsui, T. , Orlacchio, A. , … Kaji, R. (2013). A new CSF1R mutation presenting with an extensive white matter lesion mimicking primary progressive multiple sclerosis. Journal of the Neurological Sciences, 334(1–2), 192–195. 10.1016/j.jns.2013.08.020 24034409

[mgg3595-bib-0024] Karle, K. N. , Biskup, S. , Schule, R. , Schweitzer, K. J. , Kruger, R. , Bauer, P. , … Schols, L. (2013). De novo mutations in hereditary diffuse leukoencephalopathy with axonal spheroids (HDLS). Neurology, 81(23), 2039–2044. 10.1212/01.wnl.0000436945.01023.ac 24198292

[mgg3595-bib-0025] Katsumoto, A. , Takeuchi, H. , Takahashi, K. , & Tanaka, F. (2018). Microglia in Alzheimer's disease: Risk factors and inflammation. Frontiers in Neurology, 9, 978 10.3389/fneur.2018.00978 30498474 PMC6249341

[mgg3595-bib-0026] Kleinfeld, K. , Mobley, B. , Hedera, P. , Wegner, A. , Sriram, S. , & Pawate, S. (2013). Adult‐onset leukoencephalopathy with neuroaxonal spheroids and pigmented glia: Report of five cases and a new mutation. Journal of Neurology, 260(2), 558–571. 10.1007/s00415-012-6680-6 23052599

[mgg3595-bib-0027] Kohler, S. , Schulz, M. H. , Krawitz, P. , Bauer, S. , Dolken, S. , Ott, C. E. , … Robinson, P. N. (2009). Clinical diagnostics in human genetics with semantic similarity searches in ontologies. American Journal of Human Genetics, 85(4), 457–464. 10.1016/j.ajhg.2009.09.003 19800049 PMC2756558

[mgg3595-bib-0028] Kohler, S. , Vasilevsky, N. A. , Engelstad, M. , Foster, E. , McMurry, J. , Ayme, S. , … Robinson, P. N. (2017). The human phenotype ontology in 2017. Nucleic Acids Research, 45(D1), D865–D876. 10.1093/nar/gkw1039 27899602 PMC5210535

[mgg3595-bib-0029] Kohler, W. , Curiel, J. , & Vanderver, A. (2018). Adulthood leukodystrophies. Nature Reviews Neurology, 14(2), 94–105. 10.1038/nrneurol.2017.175 29302065 PMC11348681

[mgg3595-bib-0030] Konno, T. , Kasanuki, K. , Ikeuchi, T. , Dickson, D. W. , & Wszolek, Z. K. (2018). CSF1R‐related leukoencephalopathy: A major player in primary microgliopathies. Neurology, 91(24), 1092–1104. 10.1212/WNL.0000000000006642 30429277 PMC6329328

[mgg3595-bib-0031] Konno, T. , Tada, M. , Tada, M. , Koyama, A. , Nozaki, H. , Harigaya, Y. , … Ikeuchi, T. (2014). Haploinsufficiency of CSF‐1R and clinicopathologic characterization in patients with HDLS. Neurology, 82(2), 139–148. 10.1212/WNL.0000000000000046 24336230 PMC3937843

[mgg3595-bib-0032] Konno, T. , Yoshida, K. , Mizuno, T. , Kawarai, T. , Tada, M. , Nozaki, H. , … Ikeuchi, T. (2017). Clinical and genetic characterization of adult‐onset leukoencephalopathy with axonal spheroids and pigmented glia associated with CSF1R mutation. European Journal of Neurology, 24(1), 37–45. 10.1111/ene.13125 27680516 PMC5215554

[mgg3595-bib-0033] Konno, T. , Yoshida, K. , Mizuta, I. , Mizuno, T. , Kawarai, T. , Tada, M. , … Ikeuchi, T. (2018). Diagnostic criteria for adult‐onset leukoencephalopathy with axonal spheroids and pigmented glia due to CSF1R mutation. European Journal of Neurology, 25(1), 142–147. 10.1111/ene.13464 28921817 PMC5741468

[mgg3595-bib-0034] Kortvelyessy, P. , Krageloh‐Mann, I. , Mawrin, C. , Heinze, H. J. , Bittner, D. , Wieland, I. , … Nestor, P. (2015). Hereditary diffuse leukoencephalopathy with spheroids (HDLS) with a novel CSF1R mutation and spinal cord involvement. Journal of the Neurological Sciences, 358(1–2), 515–517. 10.1016/j.jns.2015.09.370 26476772

[mgg3595-bib-0035] Li, J. , Chen, K. , Zhu, L. , & Pollard, J. W. (2006). Conditional deletion of the colony stimulating factor‐1 receptor (c‐fms proto‐oncogene) in mice. Genesis, 44(7), 328–335. 10.1002/dvg.20219 16823860

[mgg3595-bib-0036] Lynch, D. S. , Jaunmuktane, Z. , Sheerin, U. M. , Phadke, R. , Brandner, S. , Milonas, I. , … Houlden, H. (2016). Hereditary leukoencephalopathy with axonal spheroids: A spectrum of phenotypes from CNS vasculitis to parkinsonism in an adult onset leukodystrophy series. Journal of Neurology, Neurosurgery and Psychiatry, 87(5), 512–519. 10.1136/jnnp-2015-310788 PMC4853550 25935893

[mgg3595-bib-0037] Lynch, D. S. , Wade, C. , Paiva, A. R. B. , John, N. , Kinsella, J. A. , Merwick, A. , … Chataway, J. (2018). Practical approach to the diagnosis of adult‐onset leukodystrophies: An updated guide in the genomic era. Journal of Neurology, Neurosurgery and Psychiatry, 1–12, 10.1136/jnnp-2018-319481 PMC6581077 30467211

[mgg3595-bib-0038] Martinez‐Muriana, A. , Mancuso, R. , Francos‐Quijorna, I. , Olmos‐Alonso, A. , Osta, R. , Perry, V. H. , … Lopez‐Vales, R. (2016). CSF1R blockade slows the progression of amyotrophic lateral sclerosis by reducing microgliosis and invasion of macrophages into peripheral nerves. Scientific Reports, 6, 25663 10.1038/srep25663 27174644 PMC4865981

[mgg3595-bib-0039] Monies, D. , Maddirevula, S. , Kurdi, W. , Alanazy, M. H. , Alkhalidi, H. , Al‐Owain, M. , … Alkuraya, F. S. (2017). Autozygosity reveals recessive mutations and novel mechanisms in dominant genes: Implications in variant interpretation. Genetics in Medicine, 19(10), 1144–1150. 10.1038/gim.2017.22 28383543

[mgg3595-bib-0040] Murray, P. J. (2018). Immune regulation by monocytes. Seminars in Immunology, 35, 12–18. 10.1016/j.smim.2017.12.005 29290545

[mgg3595-bib-0041] Nandi, S. , Gokhan, S. , Dai, X. M. , Wei, S. , Enikolopov, G. , Lin, H. , … Stanley, E. R. (2012). The CSF‐1 receptor ligands IL‐34 and CSF‐1 exhibit distinct developmental brain expression patterns and regulate neural progenitor cell maintenance and maturation. Developmental Biology, 367(2), 100–113. 10.1016/j.ydbio.2012.03.026 22542597 PMC3388946

[mgg3595-bib-0042] Olmos‐Alonso, A. , Schetters, S. T. , Sri, S. , Askew, K. , Mancuso, R. , Vargas‐Caballero, M. , … Gomez‐Nicola, D. (2016). Pharmacological targeting of CSF1R inhibits microglial proliferation and prevents the progression of Alzheimer's‐like pathology. Brain, 139(3), 891–907. 10.1093/brain/awv379 26747862 PMC4766375

[mgg3595-bib-0043] Oosterhof, N. , Kuil, L. E. , van der Linde, H. C. , Burm, S. M. , Berdowski, W. , van Ijcken, W. F. J. , … van Ham, T. J. (2018). Colony‐stimulating factor 1 receptor (CSF1R) regulates microglia density and distribution, but not microglia differentiation in vivo. Cell Reports, 24(5), 1203–1217, e1206. 10.1016/j.celrep.2018.06.113 30067976

[mgg3595-bib-0044] Pfirrmann, T. , Emmerich, D. , Ruokonen, P. , Quandt, D. , Buchen, R. , Fischer‐Zirnsak, B. , … Villavicencio‐Lorini, P. (2015). Molecular mechanism of CHRDL1‐mediated X‐linked megalocornea in humans and in *Xenopus* model. Human Molecular Genetics, 24(11), 3119–3132. 10.1093/hmg/ddv063 25712132

[mgg3595-bib-0045] Pridans, C. , Sauter, K. A. , Baer, K. , Kissel, H. , & Hume, D. A. (2013). CSF1R mutations in hereditary diffuse leukoencephalopathy with spheroids are loss of function. Scientific Reports, 3, 3013 10.1038/srep03013 24145216 PMC3804858

[mgg3595-bib-0046] Pridans, C. , Raper, A. , Davis, G. M. , Alves, J. , Sauter, K. A. , Lefevre, L. , … Hume, D. A. (2018). Pleiotropic Impacts of macrophage and microglial deficiency on development in rats with targeted mutation of the Csf1r locus. Journal of Immunology, 201(9), 2683–2699. 10.4049/jimmunol.1701783 PMC6196293 30249809

[mgg3595-bib-0047] Prinz, M. , & Priller, J. (2014). Microglia and brain macrophages in the molecular age: From origin to neuropsychiatric disease. Nature Reviews Neuroscience, 15(5), 300–312. 10.1038/nrn3722 24713688

[mgg3595-bib-0048] Quandt, D. , Fiedler, E. , Boettcher, D. , Marsch, W. , & Seliger, B. (2011). B7–h4 expression in human melanoma: Its association with patients' survival and antitumor immune response. Clinical Cancer Research, 17(10), 3100–3111. 10.1158/1078-0432.CCR-10-2268 21378130

[mgg3595-bib-0049] Rademakers, R. , Baker, M. , Nicholson, A. M. , Rutherford, N. J. , Finch, N. , Soto‐Ortolaza, A. , … Wszolek, Z. K. (2012). Mutations in the colony stimulating factor 1 receptor (CSF1R) gene cause hereditary diffuse leukoencephalopathy with spheroids. Nature Genetics, 44(2), 200–205. 10.1038/ng.1027 PMC3267847 22197934

[mgg3595-bib-0050] Sampath, P. , Moideen, K. , Ranganathan, U. D. , & Bethunaickan, R. (2018). Monocyte subsets: Phenotypes and function in tuberculosis infection. Frontiers in Immunology, 9, 1726 10.3389/fimmu.2018.01726 30105020 PMC6077267

[mgg3595-bib-0051] Sasaki, A. (2017). Microglia and brain macrophages: An update. Neuropathology, 37(5), 452–464. 10.1111/neup.12354 27859676

[mgg3595-bib-0052] Schroeter, M. L. , Vogt, B. , Frisch, S. , Becker, G. , Seese, A. , Barthel, H. , … Sabri, O. (2011). Dissociating behavioral disorders in early dementia‐An FDG‐PET study. Psychiatry Research, 194(3), 235–244. 10.1016/j.pscychresns.2011.06.009 22044532

[mgg3595-bib-0053] Schwarz, J. M. , Cooper, D. N. , Schuelke, M. , & Seelow, D. (2014). MutationTaster2: Mutation prediction for the deep‐sequencing age. Nature Methods, 11(4), 361–362. 10.1038/nmeth.2890 24681721

[mgg3595-bib-0054] Serrano, L. , Neira, J. L. , Sancho, J. , & Fersht, A. R. (1992). Effect of alanine versus glycine in alpha‐helices on protein stability. Nature, 356(6368), 453–455. 10.1038/356453a0 1557131

[mgg3595-bib-0055] Sundal, C. , & Wszolek, Z. K. (1993). CSF1R‐related adult‐onset leukoencephalopathy with axonal spheroids and pigmented Glia In Adam M. P., Ardinger H. H., Pagon R. A., Wallace S. E., Bean L. J. H., Stephens K., & Amemiya A. (Eds.), GeneReviews((R)) (pp. 1–17). Seattle, WA: University of Washington.

[mgg3595-bib-0056] Sundal, C. , Baker, M. , Karrenbauer, V. , Gustavsen, M. , Bedri, S. , Glaser, A. , … Andersen, O. (2015). Hereditary diffuse leukoencephalopathy with spheroids with phenotype of primary progressive multiple sclerosis. European Journal of Neurology, 22(2), 328–333. 10.1111/ene.12572 25311247 PMC4289423

[mgg3595-bib-0057] Sundal, C. , Lash, J. , Aasly, J. , Oygarden, S. , Roeber, S. , Kretzschman, H. , … Wszolek, Z. K. (2012). Hereditary diffuse leukoencephalopathy with axonal spheroids (HDLS): A misdiagnosed disease entity. Journal of the Neurological Sciences, 314(1–2), 130–137. 10.1016/j.jns.2011.10.006 22050953 PMC3275663

[mgg3595-bib-0058] Tada, M. , Konno, T. , Tada, M. , Tezuka, T. , Miura, T. , Mezaki, N. , … Kakita, A. (2016). Characteristic microglial features in patients with hereditary diffuse leukoencephalopathy with spheroids. Annals of Neurology, 80(4), 554–565. 10.1002/ana.24754 27490250

[mgg3595-bib-0059] Tap, W. D. , Wainberg, Z. A. , Anthony, S. P. , Ibrahim, P. N. , Zhang, C. , Healey, J. H. , … Bollag, G. (2015). Structure‐guided blockade of CSF1R kinase in tenosynovial giant‐cell tumor. New England Journal of Medicine, 373(5), 428–437. 10.1056/NEJMoa1411366 26222558

[mgg3595-bib-0060] Vihinen, M. (2013). Guidelines for reporting and using prediction tools for genetic variation analysis. Human Mutation, 34(2), 275–282. 10.1002/humu.22253 23169447

[mgg3595-bib-0061] Wider, C. , Van Gerpen, J. A. , DeArmond, S. , Shuster, E. A. , Dickson, D. W. , & Wszolek, Z. K. (2009). Leukoencephalopathy with spheroids (HDLS) and pigmentary leukodystrophy (POLD): A single entity? Neurology, 72(22), 1953–1959. 10.1212/WNL.0b013e3181a826c0 19487654 PMC2843560

[mgg3595-bib-0062] Wong, K. L. , Tai, J. J. , Wong, W. C. , Han, H. , Sem, X. , Yeap, W. H. , … Wong, S. C. (2011). Gene expression profiling reveals the defining features of the classical, intermediate, and nonclassical human monocyte subsets. Blood, 118(5), e16–31. 10.1182/blood-2010-12-326355 21653326

[mgg3595-bib-0063] Ziegler‐Heitbrock, L. (2015). Blood monocytes and their subsets: Established features and open questions. Frontiers in Immunology, 6, 423 10.3389/fimmu.2015.00423 26347746 PMC4538304

