# Supplementary material for: Functional characterization of a novel CSF1R mutation causing hereditary diffuse leukoencephalopathy with spheroids
Source: Mol Genet Genomic Med. 2019 Feb 6;7(4):e00595. doi: 10.1002/mgg3.595 (PMC6465730; doi:10.1002/mgg3.595)
Supplement: Supplementary file 1 [file MGG3-7-na-s001.docx]

**Supporting information**

**
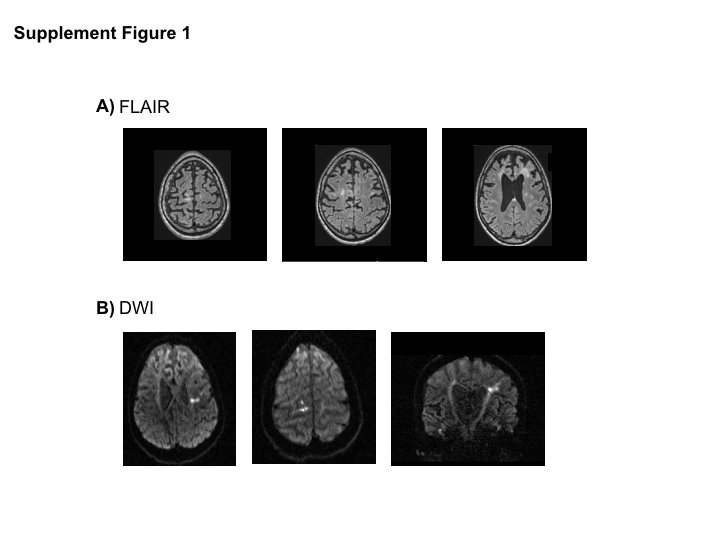
**

**Supplementary figure 1. Additional follow-up MRI of the brain.** (A) Asymmetrical diffuse white matter hyperintensities most pronounced in the frontal lobes depicted on FLAIR images. (B) Diffusion weighted imaging (DWI) revealing various isolated spots of hyperintense signal on B1000 images as sign of diffusion restriction and characteristic imaging feature for HDLS.

**
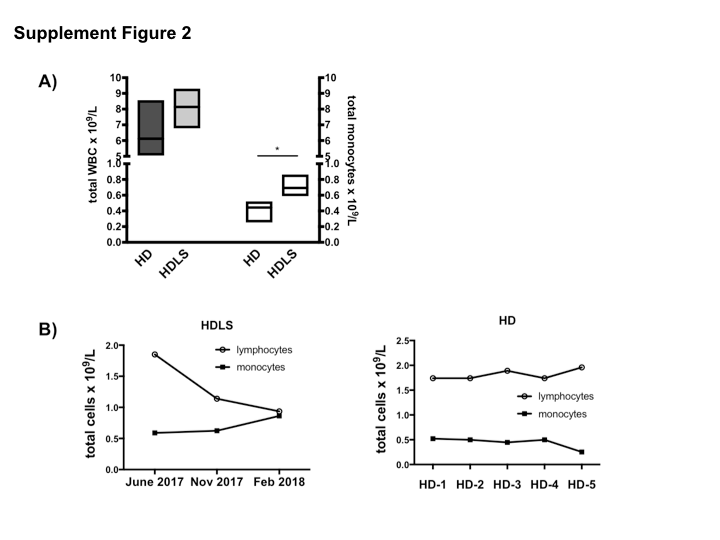
**

**Supplementary figure 2. Immune status in HDLS patient over time.** Gender and age matched healthy donors (HD) were used for comparison (n=5). HDLS patient blood was obtained at three time points in June and November 2017 and February 2018. Whole blood was analyzed by a hematology analyzer. **(A)** Total white blood cells (WBC) and total monocyte count is given. **(B)** Total monocyte and lymphocyte counts are given over time (HDLS) or single time points (HD). Floating bars from Min to Max, line at mean are given in A) and significance refers to p< 0.05.

**Supplementary material**

**List of oligonucleotide primers used for *CSF1R* gene sequencing**

**
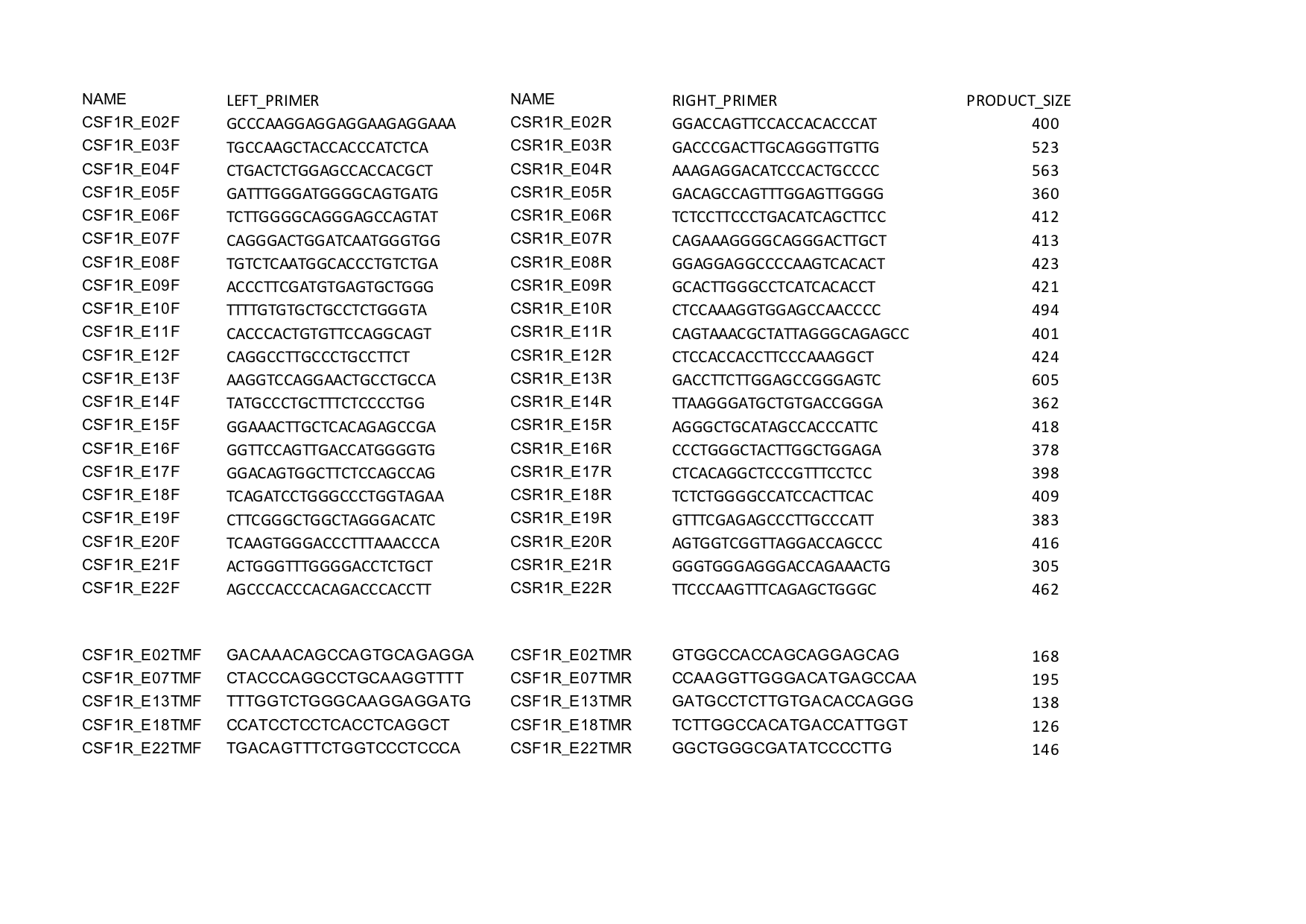
**

**Additional information according to publication guidelines for prediction method (Vihinen, 2013)**

1. The method has to be appropriate to the task, before using prediction results, understand the principle of the method, its use, limitations, and applications:

MutationTaster employs a Bayes classifier to eventually predict the disease potential of an alteration. The Bayes classifier is fed with the outcome of all tests and the features of the alterations and calculates probabilities for the alteration to be either a *disease mutation* or a harmless *polymorphism*.

Prediction of all types of sequence alterations. InDel alterations are limited to 12 bp.

1. The applied method should have proven performance:

The frequencies of all single features for known disease mutations/polymorphisms were studied in a large training set composed of >390,000 known disease mutations from HGMD Professional and >6,800,000 harmless SNPs and Indel polymorphisms from the 1000 Genomes Project (TGP).

Performance using 1000 genomes and ClinVar database:

sensitivity 93.3%, specificity 87.1%, accuracy 90.2%

1. Report method details:

Citation: Schwarz, J. M., Cooper, D. N., Schuelke, M., & Seelow, D. (2014). MutationTaster2: mutation prediction for the deep-sequencing age. Nat Methods, 11(4), 361-362. doi:10.1038/nmeth.2890

URL: http://www.mutationtaster.org

Version: MutationTaster2

Used parameters:

NCBI 37 / Ensembl 69

Probability value close to 1 indicates a high 'security' of the prediction

PhyloP/phastCons determine the grade of conservation based on the multiple alignment of genome sequences of 46 different species.

PhyloP values between -14 and +6, separately measures conservation at individual columns

PhastCons values vary between 0 and 1, the closer the value is to 1, the more probable the nucleotide is conserved, considers not just each individual alignment column, but also its flanking columns

Amino acid changes score considers the physico-chemical characteristics and may range from 0.0 to 215

Program options: Single query function by sequence input

1. User-generated multiple sequence alignment:


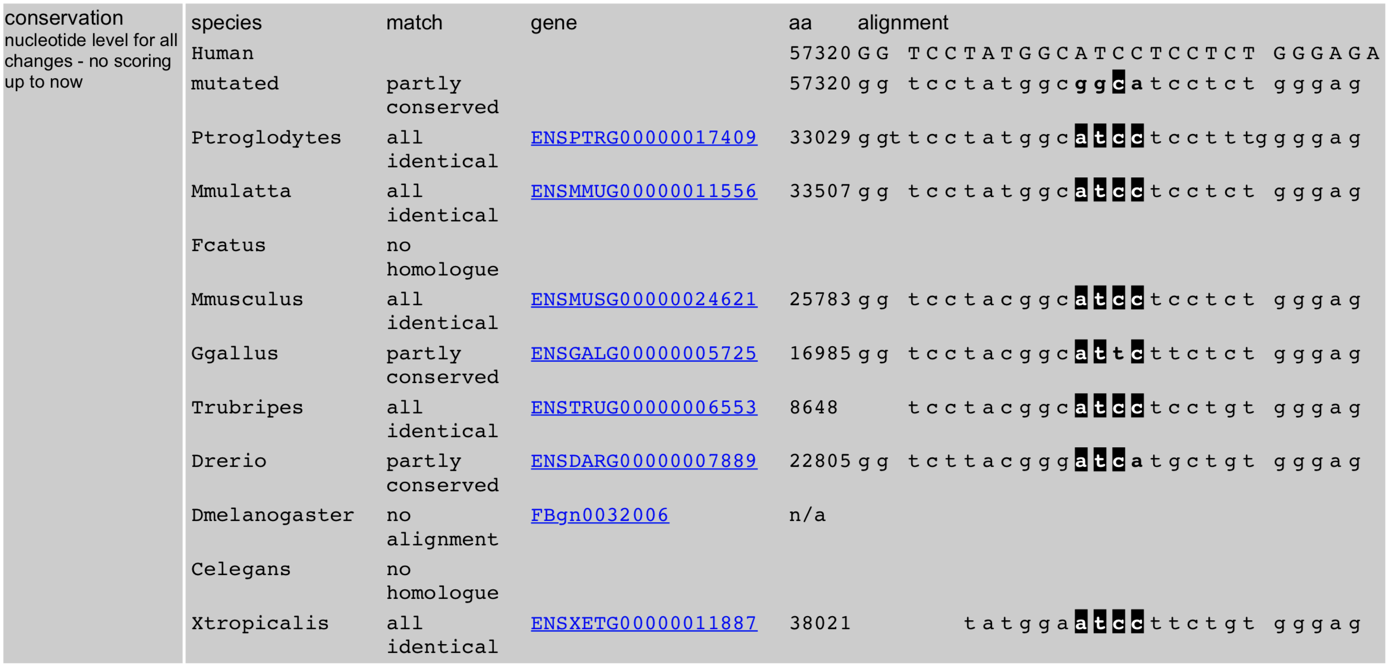


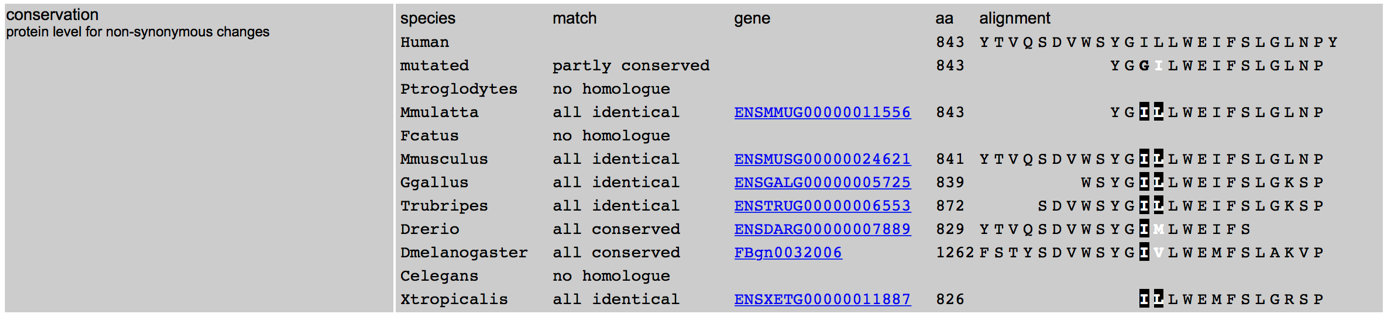


1. Include *P* values, confidence intervals, and all other reliability measures provided:


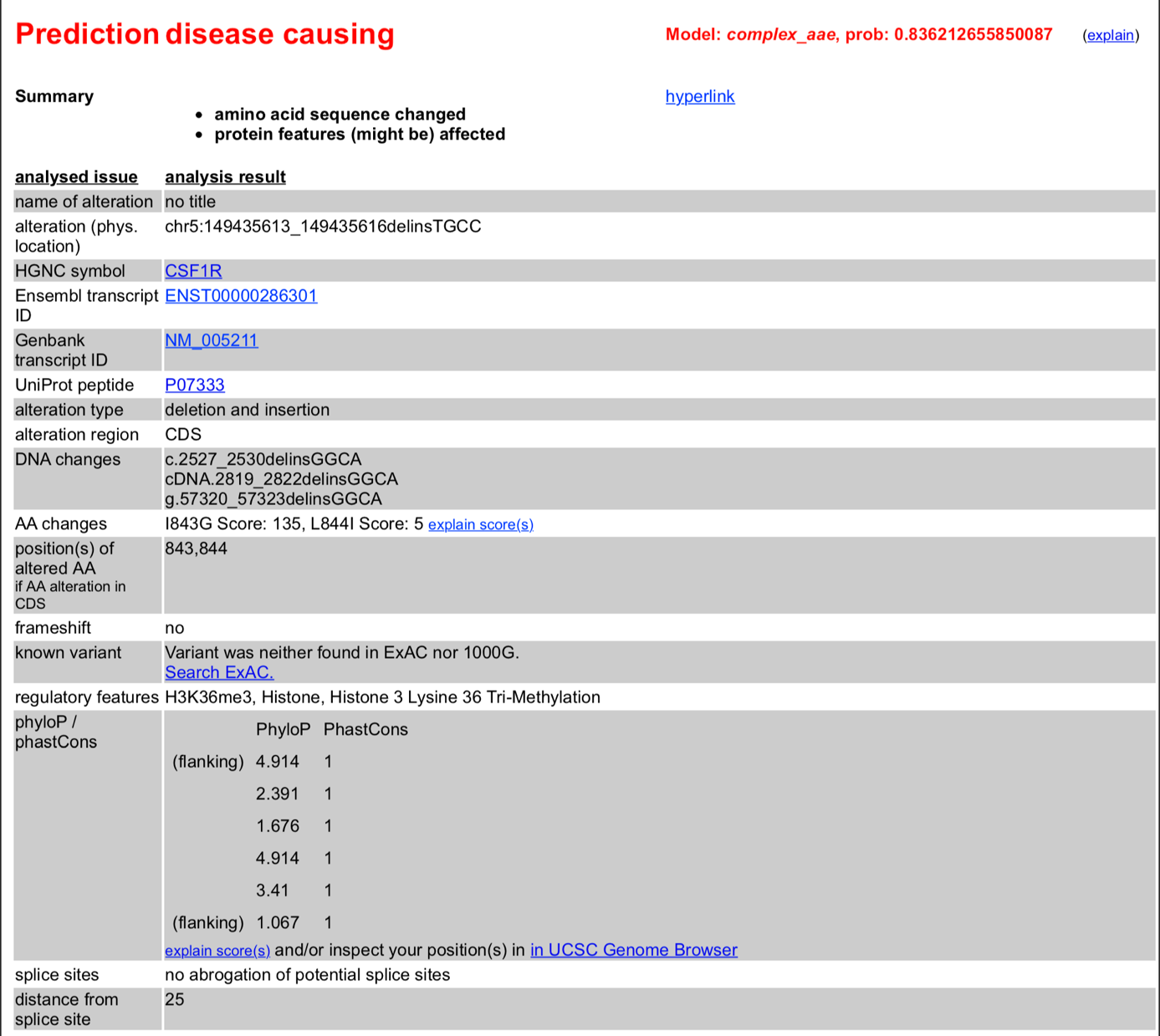


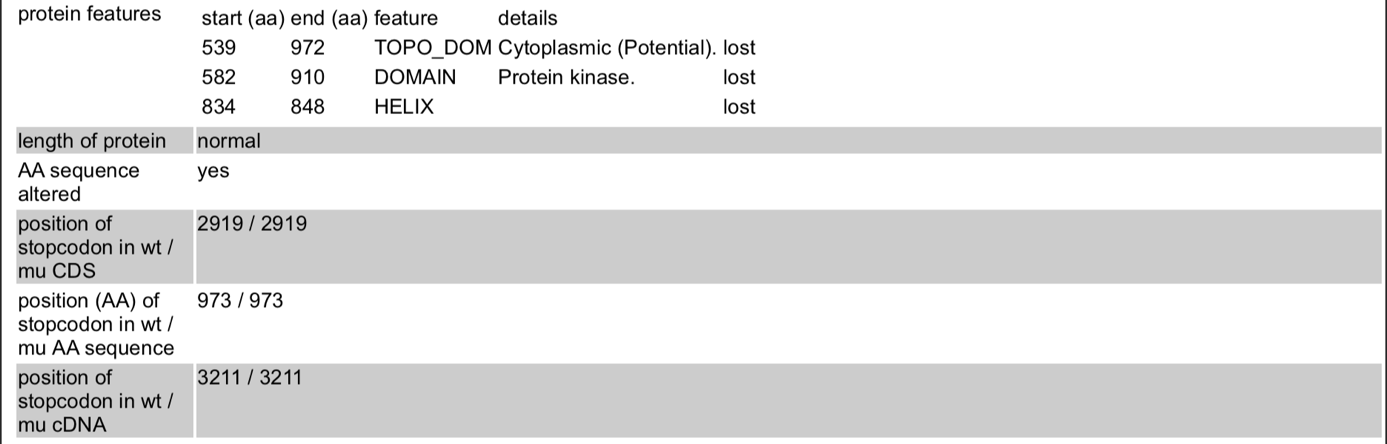


1. Be careful with data interpretation:

Additional protein structure analysis using PDB data and PyMOL

Additional functional analyses of the variant on protein level by FACS

Additional genotype-phenotype correlation by follow-up brain imaging studies

1. It may be beneficial to use several methods (if available):

SIFT and PolyPhen-2 can only handle single amino acid substitutions.

PROVEAN (URL: <http://provean.jcvi.org/index.php>)


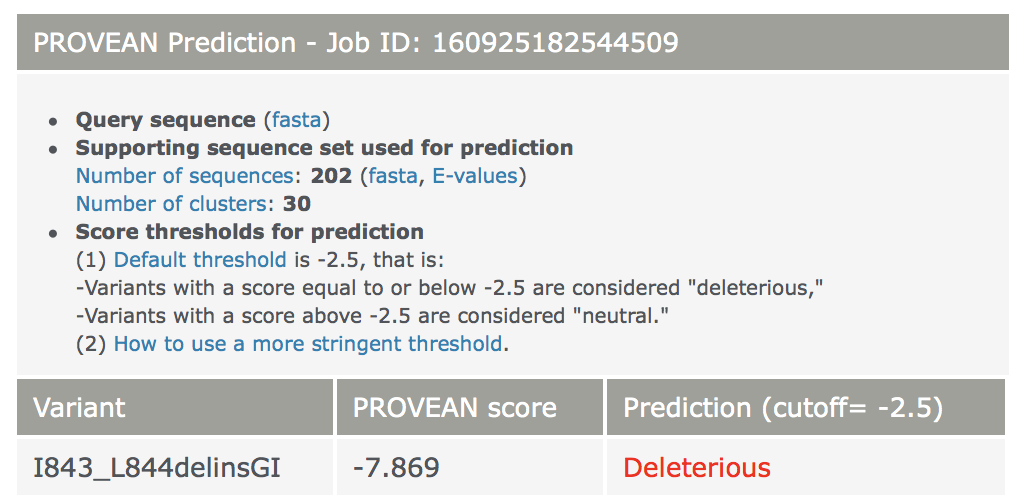


Citation: Choi, Y., & Chan, A. P. (2015). PROVEAN web server: a tool to predict the functional effect of amino acid substitutions and indels. Bioinformatics, 31(16), 2745-2747. doi:10.1093/bioinformatics/btv195
